# Differences of Corneal Biomechanics Among Thin Normal Cornea, Forme-Fruste Keratoconus, and Cornea After SMILE

**DOI:** 10.3389/fbioe.2022.861924

**Published:** 2022-05-13

**Authors:** Di Zhang, Lei Tian, Haixia Zhang, Yan Zheng, Caiyun Fu, Changbin Zhai, Ying Jie, Lin Li

**Affiliations:** ^1^ School of Biomedical Engineering, Capital Medical University, Beijing, China; ^2^ Beijing Key Laboratory of Fundamental Research on Biomechanics in Clinical Application, Capital Medical University, Beijing, China; ^3^ Beijing Advanced Innovation Center for Big Data-based Precision Medicine, Capital Medical University, Beijing, China; ^4^ Beijing Ophthalmology and Visual Sciences Key Laboratory, Beijing Institute of Ophthalmology, Beijing Tongren Eye Center, Beijing Tongren Hospital, Capital Medical University, Beijing, China; ^5^ Beijing Advanced Innovation Center for Big Data-Based Precision Medicine, Beijing Tongren Hospital, Beihang University and Capital Medical University, Beijing, China; ^6^ Beijing Ophthalmology and Visual Sciences Key Laboratory, Beijing Tongren Eye Center, Beijing Tongren Hospital, Capital Medical University, Beijing, China

**Keywords:** thin normal cornea, FFKC, post-SMILE, corneal biomechanicans, CorVis ST

## Abstract

**Background:** To compare the corneal biomechanics of thin normal cornea (TNC) with thinnest corneal thickness (TCT) (≤500 µm), forme-fruste keratoconus (FFKC) and cornea after small incision lenticule extraction (Post-SMILE) had their central corneal thickness (CCT) matched by Corneal Visualization Scheimpflug Technology (Corvis ST).

**Methods:** CCT were matched in 23 eyes with FFKC, 23 eyes by SMILE in 3 months post-operatively, and 23 TNC eyes. The differences in corneal biomechanics by Corvis ST among the three groups were compared.

**Results:** There was no significant difference in CCT among the three groups, and the biomechanically corrected intraocular pressure (bIOP) did not differ significantly among the three groups (all *p* > 0.05). There were significant differences in most DCR parameters between pre- and post-operatively (all *p* < 0.05). Compared with TNC, the values of corneal deflection amplitude during the first applanation (A1DA), length at the first applanation (A1L), corneal deflection amplitude during the second applanation (A2DA), and maximum deformation amplitude (DA) decreased in 3 months after SMILE (all *p* < 0.05), these values increased in the FFKC (all *p* < 0.05).

**Conclusion:** The majority of the DCR parameters were different among the three groups. The parameters A1DA, A1L, A2DA, and DA may be different between TNC and Post-SMILE, TNC and FFKC, and Post-SMILE and FFKC.

## Introduction

Small incision lenticule extraction (SMILE) has become one of the refractive surgery selected by patients and surgeons because of its good safety, stability, and post-operative effect (Vestergaard et al., 2014; Qin et al., 2018; Sánchez-González and Alonso-Aliste, 2019; Xia et al., 2020). The corneal morphology experiences a series of changes due to the removing part of the corneal tissue. The maintenance of post-operative corneal morphology relates to corneal biomechanical properties and intraocular pressure (IOP).

At present, corneal visualization Scheimpflug technology (Corvis ST) has been widely used to understand corneal biomechanical properties through analysis of the recorded dynamic corneal response (DCR) parameters. Several previous studies showed the changes of DCR parameters after SMILE (Cao et al., 2020; Wu et al., 2020). Takeing the effects of thinner central corneal thickness (CCT) (Vinciguerra et al., 2016; Miki et al., 2017) after SMILE on DCR parameters into account there may be misunderstandings about the interpretation of the data recorded after SMILE.

To this end, the study aimed to compare the DCR parameters of the corneas from myopic patients who underwent SMILE (Post-SMILE), forme-fruste keratoconus (FFKC) patients, and thin normal cornea (TNC) subjects with CCT-matched. This will contribute to the understanding of corneal biomechanical properties after SMILE.

## Methods

### Subjects

**FIGURE 1:**
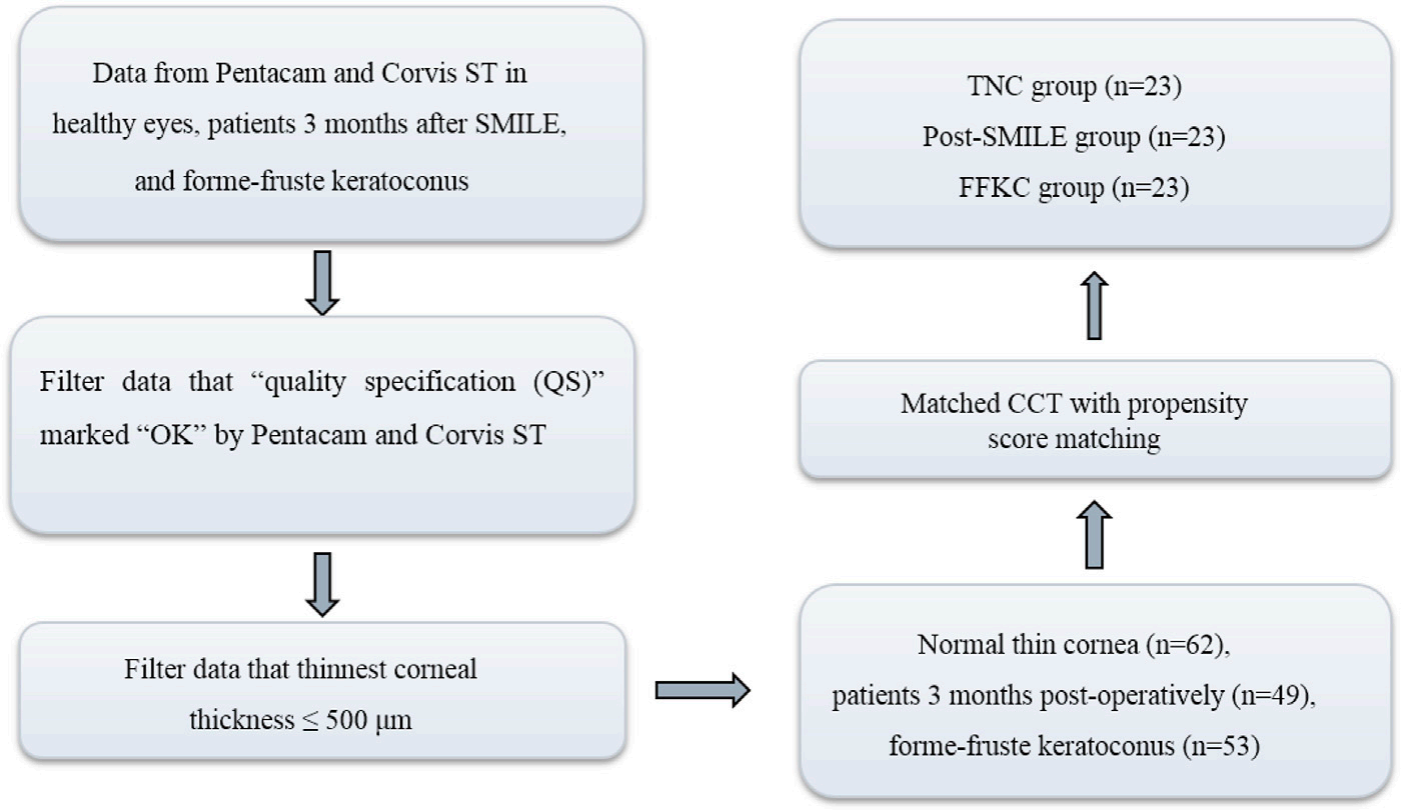
Standard flowchart.

This was a retrospective study that included 23 patients (23 eyes) who underwent SMILE (Post-SMILE group), 23 normal subjects (23 eyes) with thin normal cornea (the TNC group), and 23 FFKC patients (FFKC group). One eye was selected randomly for analysis. Each group included 23 eyes with CCT matched. The flowchart of this study was shown in Figure 1. The institutional review board of the Beijing Tongren Hospital (Beijing, China) approved this study. All participants signed an informed consent form in accordance with the tenets of the Declaration of Helsinki.

All patients who underwent SMILE at Beijing Tongren Hospital from September 2020 to January 2021. Healthy subjects and FFKC patients met the following criteria: Thinnest corneal thickness (TCT) range: ≤500 µm (Esporcatte et al., 2020); all participants had no history of corneal or ocular surgery, or trauma or systemic diseases that might affect the eye. Before the examination, if any, they had no soft contact lens utilization within 2 weeks, or had abandoned rigid contact lenses at least 3 months. The diagnostic criteria of FFKC had been described in previous studies (Tian et al., 2021).

### Ocular Examination and Collection of the Parameters

A comprehensive ocular examination was performed on the eyes of all subjects, including slit-lamp microscopy, fundus examination, tomographic measurements using the corneal Scheimpflug tomography (Pentacam HR; Oculus; Optikgeräte GmbH, Wetzlar, Germany), and biomechanical examination using the corneal visualization Scheimpflug technology (Corvis ST; Oculus; Optikgeräte GmbH, Wetzlar, Germany, software version: 1.5r1902). The patients in Post-SMILE group were measured pre-operatively, and 3 months after SMILE. Only the “quality specification (QS)” marked “OK” by Pentacam and Corvis ST were considered for further analysis and processing.

**FIGURE 2:**
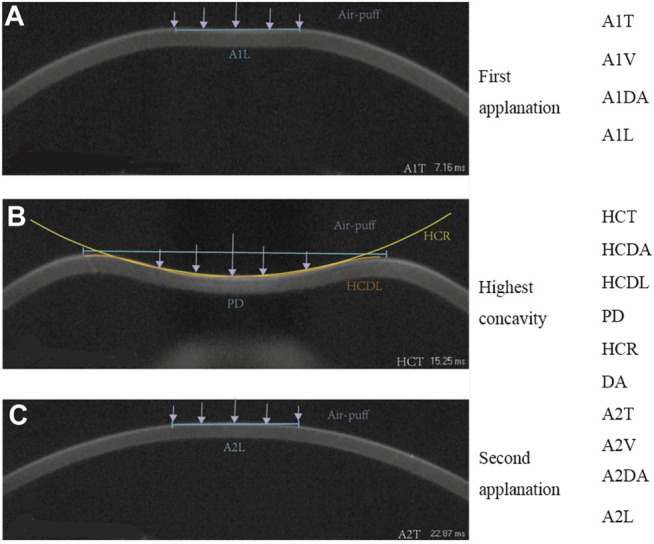
The output parameters of Corvis ST, first applanation **(A)**, highest concavity **(B)**, second applanation **(C)**.

During the test of Corvis ST, the cornea will go through the process from the initial position to the first applanation, the first applanation to the highest concavity, the highest concavity to the second applanation and return to the initial position. The parameters evaluated in the analysis are detailed in Table 1. The typical states of cornea in Corvis ST test were shown in Figure 2. The collected Corvis ST parameters are recorded as DCR.

**TABLE 1:** Corvis ST parameters of eyes by group.

Parameters	Means
A1T	Time from starting until the first applanation
A1V	Velocity of the corneal apex during the first applanation
A1DA	Corneal deflection amplitude during the first applanation
A1L	Length at the first applanation
A2T	Time from starting until the second applanation
A2V	Velocity of the corneal apex during the second applanation
A2DA	Corneal deflection amplitude during the second applanation
A2L	Length at the second applanation
HCT	Time from the measurement beginning to the moment of reaching the highest concavity
HCDA	Corneal deflection amplitude at the moment of the highest corneal concavity
HCDL	Highest concavity deflection length
PD	Peak distance at the highest concavity
HCR	Central concave curvature at highest concavity
DA	Maximum deformation amplitude
ARTh	Ambrósio relational thickness to the horizontal profile
DAR1	Deflection amplitude ratio maximal (1 mm)
DAR2	Deflection amplitude ratio maximal (2 mm)
SPA1	Stiffness parameter at the first applanation
CBI	Corneal biomechanical index
bIOP	Biomechanically corrected IOP

### Surgical Technique

The pre-operative design and surgeries were performed by the same surgeon (CB. Z.) using a repetition rate of 500 kHz VisuMax femtosecond laser system (Carl Zeiss Meditec, Jena, Germany). In all cases, the thickness of the cap was 110 or 120 μm, and the cap diameter was 7.5 mm, the lenticule diameter was 6.5 mm. All side-cut angles were 90° at a position of 120°, and incision width was 2 mm. The patients were treated with conventional anti-inflammatory drugs within 1 month post-operatively.

### Statistical Analysis

All analyses were performed using SPSS (version 23.0, IBM Corporation, Armonk, NY) and R (version 3.6.3, R Core Team). The data was tested for normality of distribution using Shapiro-Wilk test, and expressed as mean ± SD or median (inter-quartile range, IQR) (95% confidence interval of difference). Normally distributed data was analyzed using the one-way analysis of variance (one-way ANOVA), and Bonferroni test was used to compare parameters between any two of the three groups. For non-normally distributed data, Kruskal-Wallis test was used to compare parameters, and Bonferroni corrected Mann-Whitney *U* test was used to compare parameters between any two groups. Paired *t* test (normally distributed data) or Wilcoxon signed rank test (non-normally distributed data) was used to compare the parameters between pre- and post-operation. Propensity score matching, a widely used method that can control multiple confounding factors (such as CCT and IOP) at the same time, was used to match the CCT among the three groups. *p* < 0.05 was considered statistically significant.

## Results

The information of eyes by group after CCT matched was shown in Table 2. Including 69 eyes of 69 individuals, 23 individuals in each group. Among the three groups, no statistical differences (all *p* > 0.05) were detected in the gender (chi-square test), bIOP, TCT (Kruskal-Wallis test for the three groups, and Bonferroni corrected Mann-Whitney *U* test for any two groups.), and CCT (one-way ANOVA for the three groups, and Bonferroni test for any two groups). However, the age in Post-SMILE group was greater than those in group TNC and group FFKC.

**TABLE 2:** Baseline information of eyes by group after CCT matched.

Parameters	TNC	Post-SMILE	FFKC	*p*
Gender (Male/Female)	11/12	7/16	10/13	0.458
Age (years)	24 ± 3 (23–26)	31 (9) (28–32)^#^	21 (9) (21–25)^&^	<0.001
CCT (μm)	488 ± 10 (484–492)	484 ± 12 (478–489)	491 ± 12 (486–496)	0.116
TCT (μm)	484 (19) (480–488)	482 ± 13 (476–487)	483 (17) (481–490)	0.559
bIOP (mmHg)	14.6 ± 2.1 (13.7–15.5)	13.3 ± 1.7 (12.5–14.0)	13.8 (2.6) (13.6–14.9)	0.086

Data is presented as mean ± SD (95% confidence interval) or median (IQR) (95% confidence interval). The *p* value was from the test among the three groups; ^#^, ^&^ represent statistically significant difference with TNC and Post-SMILE, and Post-SMILE and FFKC, respectively.

Table 3 summarizes the values of the DCR parameters of all individuals. Overall, there were significant differences in the DCR between Pre- and Post-SMILE groups (all *p* < 0.05), except for A2V, A2L, HCT and HCDL. 6/19, 6/19, and 8/19 DCR parameters were significantly different between the TNC and Post-SMILE, between the TNC and FFKC, between the Post-SMILE and FFKC, respectively (all *p* < 0.05). Statistically differences were detected in SPA1 between Pre- and Post-SMILE, and between Post-SMILE and FFKC (*p* < 0.05).

**TABLE 3:** Corvis ST parameters of eyes by group.

Parameters	TNC	Pre-SMILE	Post-SMILE	FFKC	*p*
A1T (ms)	7.282 ± 0.251 (7.173 to 7.390)	7.754 ± 0.208 (7.644 to 7.843)	7.259 (0.362)(7.149 to 7.325)^§,c^	7.039 (0.294)(6.996 to 7.163)^#,&^	0.005^b^
A1V (m/s)	0.148 (0.018) (0.132 to 0.153)	0.149 ± 0.014 (0.143 to 0.155)	0.159 ± 0.012 (0.153 to 0.164)^*,§,c^	0.167 ± 0.015 (0.160 to 0.173)^#^	<0.001^b^
A2T (ms)	21.787 ± 0.361 (21.631 to 21.943)	22.003 ± 0.293 (21.876 to 22.130)	22.322 ± 0.419 (22.140 to 22.503)^*,§,d^	22.095 ± 0.315 (21.959 to 22.231)^#^	<0.001^a^
A2V (m/s)	−0.288 (0.047)(−0.289 to −0.262)	−0.269 (0.031)(−0.275 to −0.261)	−0.271 ± 0.018 (−0.279 to −0.263)^d^	−0.299 ± 0.040 (−0.316 to −0.282)^&^	0.005^b^
A2L (mm)	2.318 (0.467)(2.075 to 2.557)	2.824 ± 0.534 (2.593 to 3.055)	2.269 (1.523)(2.340 to 3.097)^c^	2.490 (1.909)(2.405 to 3.188)	0.284^b^
HCT (ms)	16.792 ± 0.494 (16.578 to 17.006)	17.401 ± 0.396 (17.230 to 17.573)	17.409 ± 0.450 (17.215 to 17.604)^*,c^	16.724 ± 0.509 (16.504 to 16.944)^&^	<0.001^a^
HCDA (mm)	0.942 ± 0.090 (0.903 to 0.981)	0.875 (0.097)(0.855 to 0.913)	0.972 ± 0.080 (0.937 to 1.006)^§,d^	1.039 ± 0.124 (0.986 to 1.093)^#^	0.005^a^
HCDL (mm)	6.409 ± 0.422 (6.227 to 6.592)	6.392 ± 0.272 (6.274 to 6.510)	6.532 ± 0.389 (6.364 to 6.701)^c^	6.862 ± 0.434 (6.674 to 7.049)^#,&^	0.001^a^
PD (mm)	5.103 ± 0.236 (5.001 to 5.205)	4.914 (0.267)(4.892 to 5.053)	5.287 ± 0.203 (5.199 to 5.375)^*,§,d^	5.343 ± 0.254 (5.233 to 5.453)^#^	0.002^a^
HCR (mm)	6.421 ± 0.585 (6.168 to 6.674)	6.817 (0.595)(6.709 to 7.371)	6.139 ± 0.448 (5.945 to 6.333)^§,d^	6.738 ± 0.586 (6.485 to 6.992)^&^	0.002^a^
ARTh	367.537 (82.236)(337.787 to 415.320)	482.189 ± 75.588 (449.503 to 514.876)	212.309 (43.007)(186.892 to 215.604)^*,§,c^	445.522 ± 124.505 (391.682 to 499.362)^&^	<0.001
DAR1	1.643 ± 0.058 (1.618 to 1.668)	1.529 ± 0.032 (1.515 to 1.543)	1.630 ± 0.041 (1.613 to 1.648)^§,c^	1.617 ± 0.038 (1.600 to 1.633)	0.165^a^
DAR2	4.859 ± 0.365 (4.701 to 5.017)	4.005 ± 0.225 (3.907 to 4.102)	5.075 ± 0.385 (4.909 to 5.242)^§,c^	4.971 ± 0.452 (4.776 to 5.167)	0.197^a^
SPA1	78.532 ± 12.191 (73.260 to 83.803)	114.063 ± 8.818 (110.250 to 117.876)	84.464 ± 13.372 (78.682 to 90.247)^§,c^	69.935 (9.844)(69.032 to 76.843)^&^	0.004^b^
CBI	0.944 (0.085)(0.830 to 0.951)	0.005 (0.016)(0.000 to 0.043)	0.999 (0.003)(0.988 to 1.002)^*,§,d^	0.699 (0.889)(0.394 to 0.743)^&^	<0.001^b^

Data is presented as mean ± SD (95% confidence interval) or median (IQR) (95% confidence interval). The *p* value is from the test among the three groups. The statistically significant differences were denoted by ^*^ between TNC and Post-SMILE, by ^#^ between TNC and FFKC, by ^&^ between Post-SMILE and FFKC, and by^§^ between Pre- and Post-SMILE; ^a^, ^b^, ^c^, and ^d^ represent one-way ANOVA, Kruskal-Wallis test, paired *t* test, and Wilcoxon signed rank test, respectively.

**FIGURE 3:**
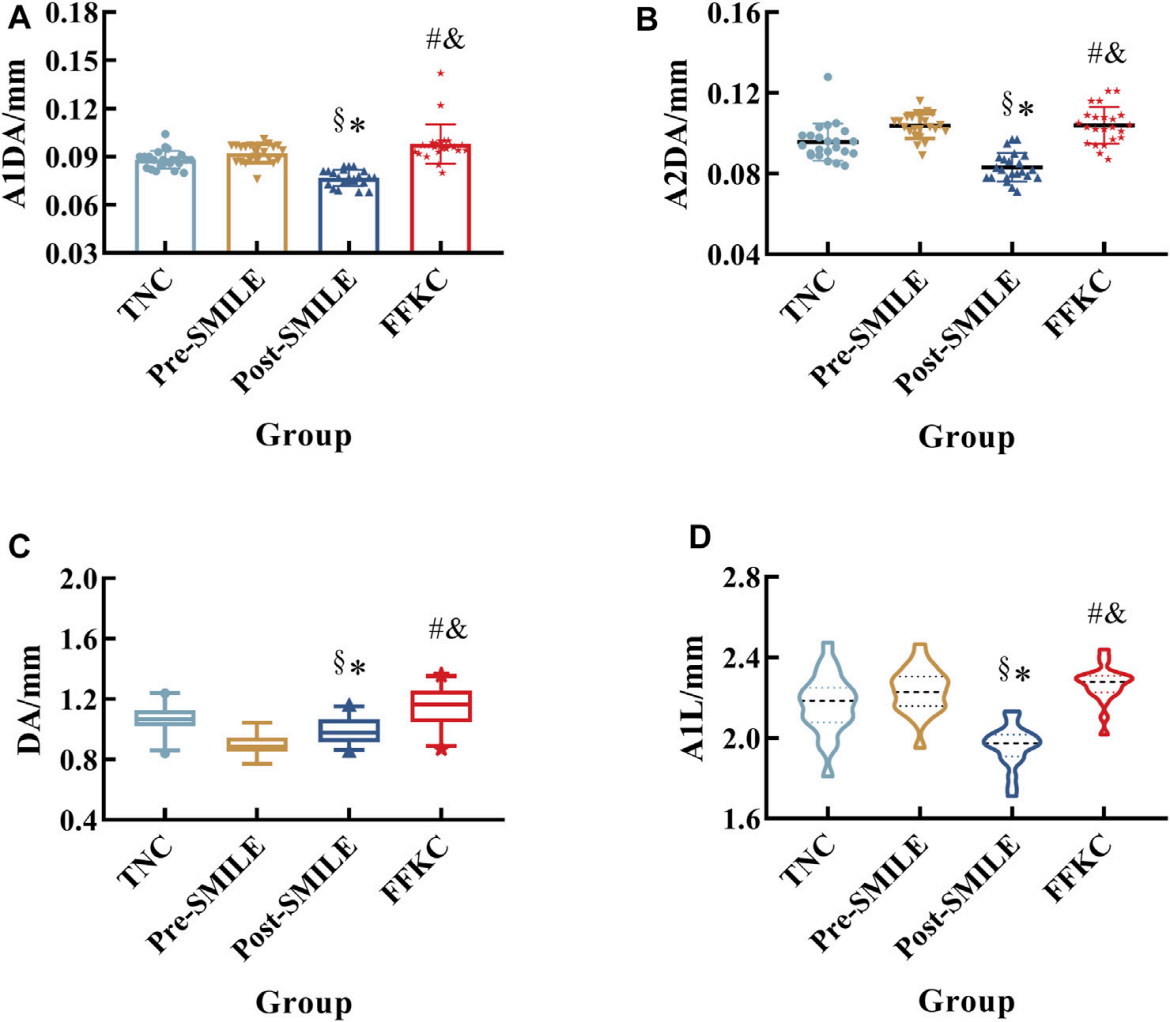
Differences among the three groups with respect to A1DA **(A)**, A2DA **(B)**, DA **(C)**, and A1L **(D)**. (*, ^#^, ^&^, and ^§^represent statistically significant differences between TNC and Post-SMILE, TNC and FFKC, Post-SMILE and FFKC, and Pre- and Post-SMILE, respectively.)

Furthermore, there were significant differences in A1DA, A1L, A2DA, and DA among the TNC, Post-SMILE, and FFKC groups. Also these differences were found between the Post-SMILE and FFKC, between the Pre- and Post-SMILE groups, respectively (all *p* < 0.05, Figure 3). Figure 3 shows that the A1DA, A2DA, A1L, and DA values in the Post-SMILE group were lowest, and that the FFKC group had higer A1DA, A2DA, A1L, and DA values compared to TNC group (all *p* < 0.05).

## Discussion

The cornea is mainly composed of corneal stroma with regularly arranged collagen fiber bundle lamellae (Mercatelli and Mattana, 2019). Corneal stroma is mainly load-bearing component of cornea. Since part of the corneal tissue is removed after corneal refractive surgery, it needs to explore whether the biomechanical properties of cornea could change when the influence of corneal thickness is discharged. This study compared the biomechanical properties of thin normal corneas, post-operation corneas, and FFKC corneas. The results showed that differences were detected in the majority of DCR parameters among TNC, Post-SMILE, and FFKC, when the CCT matched. The parameters A1DA, A1L, A2DA, and DA were significantly different between the TNC and Post-SMILE, between the TNC and FFKC, between the Post-SMILE and FFKC, and between pre- and post-operation, respectively.

The data showed that there were differences of DCR parameters between pre- and post-operation. Smaller A1L, A1T, and HCR, while larger A2T, PD, DA, and DAR (DAR1/DAR2) post-operatively compared with pre-operation (Table 3; Figure 3) were consistent with previous studies (Fernández et al., 2017; Cao et al., 2020; Wu et al., 2020). Lager A1DA, A2DA, DA, and A1L in FFKC than TNC (Figure 3) were consistent with our previous results (Tian et al., 2021) when the CCT matched. This study shows that the values of 16 and 10 DCR parameters out of the 19 involved parameters, such as A1DA, A2DA, DA, and A1L had significant differences among the three groups, and between any two groups, respectively. The parameters A1DA, DA and A2DA represent the corneal deflection amplitude at the first, maximum deformation amplitude, highest concavity and second applanation, and A1L is the length at the first applanation. In theory, under the same external loads (air-puff and IOP), the corneas without geometric differences (CCT matched, differences of curvature ignored) should have little differences in deformation. Studies (Huseynova et al., 2014; Wang et al., 2017; Sedaghat et al., 2020) showed that A1L was correlated with CCT and IOP, but not with age and myopia. Our results showed that A1L in Post-SMILE, TNC and FFKC increased in turn (Figure 2D), where CCT, TCT, and bIOP were matched with the same baseline level among the three groups (Table 2). We tended to attribute the differences of A1L among the three groups to the differences in corneal biomechanical properties. Based on the above analyses, the corneas in the three groups may show some differences on biomechanical properties.

The corneal mechanical properties may have variations after SMILE. The data showed that smaller A1DA, A1L, and A2DA, while larger DA in Post-SMILE group than Pre-SMILE group (Figure 3). And after CCT matching, the values of A1DA, A1L, A2DA and DA gave their the largest in the FFKC group, the smallest in the Post-SMILE group, moderate in the TNC group. Since the distribution of the applied air-puff is the same during Corvis ST tests, and the bIOP among the three groups was basically maintained at the same level, these variations of DCR parameters suggest that there may be differences in corneal biomechanics among the TNC group, Post-SMILE, and FFKC.

It can be explained from the micro level why the corneal mechanical properties are different between Post-SMILE and TNC. The corneal stromal cells would be adjusted (Dong et al., 2014), and corneal epithelial will be remolded (Luft et al., 2016; Romito et al., 2020) by observing the microstructure of cornea after SMILE. The cell proliferation reached the peak at 1 week after SMILE, and the transforming growth factor-β1 (TGF-β1), which can promote the proliferation of corneal stromal cells (Ljubimov and Saghizadeh, 2015) and may cause tissue fibrosis (Wang et al., 2011), was still at a high level within 1 month after SMILE by animal experimental observation (Liu et al., 2020). Furthermore, the remodeling of corneal extracellular matrix is affected by the change of mechanical environment (Du et al., 2017), and then affect the migration and metabolism of keratocytes, which may affect the stability of corneal tissue. This may lead to the corneal mechanical properties are different between Post-SMILE and TNC.

However, if we aim to know the characteristics of corneal biomechanical properties of each group, we need to further analysis biomechanical parameters, such as, elastic modulus and nonlinear elasticity, based on the relationship between DCR parameters and corneal biomechanical properties (not established yet) or the established mechanical models using the data output by Corvis ST (Boszczyk et al., 2017; Jannesari et al., 2019; Qin et al., 2019; Zhang et al., 2021), or direct measuring by optical correlation elastic imaging (Singh et al., 2018; Zhou et al., 2019; De Stefano et al., 2020; Chong and Dupps, 2021; Lan et al., 2021), Brillouin microscopy (Yun and Chernyak, 2018; Shao and Eltony, 2019; Chong and Dupps, 2021). In this study, we did not consider the viscous processes and a plastic deformation of the stroma during the surgical procedure. It is possibly needed to study in the future.

SMILE and FS-LASIK are two of safe and widely applied procedures for corneal refractive surgery. A large number of studies have investigated the biomechanical properties of corneas after FS-LASIK and SMILE *via* Ocular Response Analyzer (ORA) testing or Corvis ST testing, and found no difference in DCR parameters, corneal hysteresis (CH) or corneal resistance factor (CRF) between SMILE and FS-LASIK *in vivo* (Sefat et al., 2016; Xia et al., 2016; Raevdal et al., 2019; Cao et al., 2020). This study explored the changes in corneal biomechanical properties after SMILE, and the biomechanical properties of normal corneas, post-operative corneas, and FFKC corneas were compared when the CCT matched. The method may be used to analyze biomechanical properties of the corneas after FS-LASIK.

There are some limitations in this study. The number of patients included was small, and further clinical studies are needed, including more samples and longer follow up time to confirm these observations. The main purpose of this study is to compare the corneal biomechanicanics of TNC, Post-SMILE, and FFKC when the CCT-matched. There is lack of stratification of data based on low, moderate, and high myopia due to the small sample size.

## Conclusion

In conclusion, the majority of DCR parameters were different among TNC, Post-SMILE, and FFKC when CCT matched. The parameters A1DA, A1L, A2DA, and DA values may be different between TNC and Post-SMILE, TNC and FFKC, and Post-SMILE and FFKC. Further observations and analyses are needed.

## Data Availability

The original contributions presented in the study are included in the article/Supplementary Material, further inquiries can be directed to the corresponding authors.
